# Management of Very Old Patients in Intensive Care Units

**DOI:** 10.14336/AD.2020.0914

**Published:** 2021-04-01

**Authors:** Xin Ding, Hui Lian, Xiaoting Wang, Chinese Critical Ultrasound Study Group

**Affiliations:** ^1^Department of Critical Care Medicine, Peking Union Medical College Hospital, Chinese Academy of Medical Sciences and Peking Union Medical College, Beijing, China.; ^2^Department of Health Care, Peking Union Medical College Hospital, Chinese Academy of Medical Sciences and Peking Union Medical College, Beijing, China.

**Keywords:** very old patients, treatment strategy, ABCCDEFGHI bundles, intensive care unit

## Abstract

The global population is aging and the demand for critical care wards increasing. Aging is associated not only with physiological and cognitive vulnerability, but also with a decline in organ function. A new topic in geriatric care is how to appropriately use critical care resources and provide the best treatment plan for very old patients (VOPs). Our special geriatric intensive care unit has admitted nearly 500 VOPs. In this review, we share our VOP treatment strategy and summarize the key points as “ABCCDEFGHI bundles.” The aim is to help intensivists to provide more comprehensive therapy for VOPs in intensive care units.

The global population is rapidly aging. By 2050, the proportion of the global population aged >60 years will have increased from 12% in 2013 to an estimated 21% [1]. The rapid expansion of the elderly population has increased the demand for critical care wards [2]. Although there is no clear consensus, patients >80 years are generally defined as “very old patients” (VOPs) in the field of critical care medicine. In the past 20 years, the number of VOPs in intensive care units (ICUs) has increased substantially and continues to grow.

Aging is a complex transition that includes physiological and cognitive vulnerability, which make the individual more prone to diseases and acute medical events, leading to further reduction in reserve capacities, loss of functional independency, and ultimately to death[3]. It has been demonstrated that frailty is an independent risk factor for mortality [4]. The Clinical Frailty Scale is currently used to assess the severity of frailty. Frailty is common among hospitalized VOPs. Measurement of frailty in this population can help doctors to increase the efficiency of clinical activities and improve the prognosis of patients at risk [5].

Aging is associated with a decline in the immune system, termed “immune senescence.” Both cellular and serological responses are affected during the aging process. Multiple changes damage the immune body cells, disrupting immune regulation [6]. In addition, chronic diseases associated with aging can also lead to mild inflammation and increased levels of circulating cytokines and pro-inflammatory markers, leading to prolonged reaction time to infection [7]. As a result, VOPs are more susceptible to infections than younger patients.

Because of these characteristics, older people are more likely to get infections and have a poor prognosis. The most common reasons for ICU admission are postoperative organ failure and sepsis [8]. Research on outcomes in critically ill patients with sepsis worldwide shows that hospital mortality increases with age. VOPs have twice the mortality as that of patients aged ≤50 years (49.3% vs. 25.2%, p < 0.05)[8]. Despite the high intensity of care and prolonged hospitalization, the total mortality of VOPs in the ICU is high. Recent observational studies have found an ICU mortality of between 15% and 25% [9]. Therefore, how to use critical care resources appropriately and provide the best treatment plan for VOPs is a new topic in the development of critical care medicine.

Our special geriatric ICU has admitted nearly 500 VOPs. In this review, we share our treatment strategy for VOPs and summarize the key points, which we call “ABCCDEFGHI bundles” (Table 1), based on recent positive results and our own experiences. We hope that these will inform future clinical practice.

## Airway: Protective reflex impairment is an important cause of aspiration pneumonia

Aspiration pneumonia is an increasingly prevalent problem in older people and is associated with high mortality [10, 11]. For patients aged ≥65 years in the United States, the incidence of aspiration pneumonia was 30.9 cases per 10,000 people, and the mortality was 11.3% in 2012, which was two times higher than in patients aged <65 years [12]. Age >65 years is an independent predictor of in-hospital mortality among patients admitted for aspiration pneumonia.

There is a pronounced decrease in the protective reflex of the airway (i.e., swallowing and cough reflexes) with age[13]. Pontoppidan et al found that the threshold of the protective airway reflex increases 6-fold from the second to the eighth and ninth decades of life, which explains why silent tracheal aspiration seems to be more frequent in older people [14]. The protective airway reflex can be further depressed by injudicious use of depressant drugs in elderly patients [15]. Furthermore, age-related changes in hyoid bone position may also contribute to reduced swallowing safety and aspiration. The sensory component of protective reflexes, which involves the sensory cortex, seems to be more vulnerable to aging than the motor component; therefore, treatments that enhance sensory nerve terminals and sensory cortical areas may be useful [16].

Although there is no consensus on standard protocols for the prevention of aspiration pneumonia, several measures should be applied. The maintenance of good oral hygiene is important and medications that affect salivary flow or cause sedation are best avoided if possible. The use of H2 blockers and proton-pump inhibitors should be minimized [17]. Tube feeding is a last resort and the use of gastrojejunostomy inserted through percutaneous endoscopic gastrostomy may be a good choice [18]. There is no evidence that any specific antibacterial agent has superior efficacy, and the use of broad-spectrum antibiotics can result in multiresistancy [19].

**Table 1:** Key points of the management of VOPs in ICU.

Airway	Protective reflex impairment is an important cause of aspiration pneumonia.
Breathing	Cardiovascular factors should be paid close attention in patients with dyspnea.
Circulation	Cardiac rhythms abnormalities, especially atrial fibrillation are often the Deteriorating factors of the circulatory system.
Comorbidities and multimorbidities	A strong predictive index of mortality.
Delirium	Monitoring and non-pharmacological prevention is more important.
Enteral nutrition	It is important especially for malnourished VOPs.
Frailty	A risk factor that should not be neglected.
Geriatrician	Comprehensive evaluation is helpful for the rehabilitation of VOPs.
Homeostasis	The cornerstone of treatment for critically ill VOPs.
Infection	Prevention and early recognition are more important.

## Breathing: Cardiovascular factors should be paid close attention in patients with dyspnea

The mortality rate for elderly patients receiving mechanical ventilation is approximately 53% [20]. Thus, the management of breathing abnormalities in elderly patients is very important. Aging is associated with changes in respiratory functions such as lung volume and gas exchange. The total lung capacity remains constant in adults, but from age 20 years, vital capacity decreases by 26 ml/year for men and 22 ml/year for women [21]. Thus, residual volume increases with age. There are changes in the ratio of residual volume and total lung capacity, forced expiratory volume in 1s, and forced vital capacity [22]. Owing to a disproportion between ventilation and blood flow, arterial oxygen pressure decreases. As lung compliance decreases with age, there is a greater tendency for airway collapse, especially in the gravity-dependent region [23]. Abnormally early airway collapse leads to both reduced abilities to generate normal expiratory pressures and increased expiratory resistance.

Dyspnea is one of the most common respiratory complaints and is frequently reported by elderly patients [24]. The estimated prevalence of dyspnea is 21% for patients aged 65 years [25] and 30% for those aged ≥80 years [26]. Dyspnea causes substantial disability and may lead to mortality [27]. There are many causes of dyspnea, including respiratory system abnormalities, cardiovascular disease, diaphragmatic dysfunction, and endocrine diseases [28]. Several studies indicate that, in addition to the focus on respiratory diseases, more attention should be paid to cardiovascular diseases [27, 29].

The aging process is a major contributor to cardiovascular system changes in older people [30, 31]. Although resting left ventricular diastolic function does not change, left ventricular diastolic function alters substantially [32]. Myocardial velocities measured using tissue doppler imaging of the mitral annulus during diastole fall progressively with age; in addition there is an increase in left ventricular diastolic stiffness that correlates with advancing age [33]. There is a steep increase in the prevalence of heart failure in older adults with normal left ventricular systolic function [34]. A significant reduction in early left ventricular filling may leave the left ventricle less distended and result in a failure of the Frank-Starling mechanism, together with an age-associated reduction in left ventricular compliance, which can easily cause increases in left atrial and left ventricular end diastolic pressure and lead to pulmonary congestion and edema. Thus, age-related changes in cardiovascular function, and the high prevalence of hypertension and coronary artery disease in the older population, combine to reduce cardiovascular reserve and increase the risk of heart failure in older adults, which is a main cause of dyspnea [35]. As 24-hour urine output also decreases with age [36], a conservative fluid management strategy should be administrated, which may attenuate the age-associated increase in ventilator-associated mortality [37].

## Circulation: Cardiac rhythms abnormalities, especially atrial fibrillation are often the deteriorating factors of the circulatory system

With aging, the heart undergoes specific changes [38]. Although resting heart rate is not affected by age, the incidence of arrhythmias increases [39-41]. At the cellular level, reduced Ca^2+^ reuptake in myocytes leads to prolongation of action potentials, and several complex changes in calcium-related ion currents and other factors slow cellular reactions responsible for controlling the periodicity of heartbeats. Furthermore, the general increase in fibrous content and the calcification process in cardiac tissue may lead to dysfunction in the atrioventricular node, and an increase in the risk of atrioventricular block [42].

Atrial fibrillation (AF) is found in approximately 3% to 4% of subjects aged >60 years, a rate 10-fold higher than that of the general adult population [40]. With age, increased myocardial stiffness leads to slower ventricle filling and reduced cardiac filling during the early diastolic phase, which increases the importance of the active filling phase during atrial contraction. Therefore, older patients with AF may have stable decompensated heart failure that is associated with increased mortality [39, 43]. The management of elderly patients with AF should focus on the presence of hemodynamic compromise, direct therapy to address the (reversible) causes of AF, control of heart rate, and prevention or treatment of heart failure and ischemia [44, 45]. Emphasis should be placed on optimizing the benefits and reducing the risks of anticoagulation in this important and expanding group of patients [46].

## Comorbidities and multimorbidities: a strong predictive index of mortality

The proportion of patients with comorbidities and the number of comorbidities (combination of additional diseases beyond an index disorder) per patient increase with age; the mean number of comorbidities per patient is 3.6 in patients aged ≥85 years [47]. The prevalence of more than one comorbid illness is substantially higher in ICU patients aged ≥65 years, and is associated with higher in-hospital [36, 48] and long-term mortality rates [49]. The most common comorbidities in the ICU are cardiovascular diseases such as hypertension and cardiac failure, respiratory diseases such as chronic obstructive pulmonary disease, diseases associated with a compromised immune system, metastatic cancer, hepatic disease, end-stage kidney disease, and hematologic malignancy [36, 50]. The Charlson comorbidity index has been validated for critically ill patients and predicts mortality.

According to one systematic review, multimorbidity (the coexistence of more than two chronic diseases) affects 55% to 98% of the elderly population. The main consequences of multimorbidity are functional impairment, poor quality of life, and high health care costs [51]. A retrospective study of 442,962 ICU patients concluded that, unlike the comorbidities mentioned above, angina, dysrhythmias, acute myocardial infarction, and drug overdose are the most common multimorbidities in the ICU. To fully evaluate a patient, a multimorbidity index (MMI) has been developed that can help to assess the patient prognosis [52].

With age, physiological changes lead to a reduction in reserve capacities. Advanced age is associated with changes in respiratory physiology (loss of elastic lung tissue, increased anteroposterior chest diameter, reduced muscle strength, and sensitivity of respiratory centers to hypoxemia and hypercapnia), leading to an increased risk of acute respiratory failure and mortality [53]. Furthermore, the proliferative capacity and the number of immune cells renewed from hematopoietic stem cells decline owing to progressive telomere shortening. This results in gradual immune dysfunction, or immune senescence. Immune senescence not only increases the risk of sepsis, but also leads to more severe infection and may also be related to higher mortality [54]. Overall, comorbidities and multimorbidities complicate disease conditions and affect outcomes in the older population.

## Delirium: Monitoring and non-pharmacological prevention is more important

Delirium is an acute disorder of attention and cognition in elderly people that is common, serious, costly, under-recognized, and often fatal [55, 56]. Nearly 30% of older patients (aged >65 years) experience delirium at some point during hospitalization, and 40% of older patients admitted to the ICU have delirium [57]. The development of delirium depends on complex inter-relationships between patient vulnerability, several predisposing factors, and exposure to noxious insults or predisposing factors. The leading risk factors are dementia or cognitive impairment, functional impairment, visual impairment, and history of alcohol misuse [58]. The gradual accumulation of permanent damage to neurons, dendrites, receptors, and microglia, and the effects of cerebrovascular disease or head trauma, can render older people susceptible to delirium when biologically stressed, especially in the presence of underlying cognitive impairment [59]. Delirium is also a risk factor for prolonged duration of mechanical ventilation, longer ICU stay, and mortality [60, 61]. Early recognition and diagnosis can improve patient prognosis. Thus, delirium monitoring should be an important routine clinical task. However, delirium is usually underdiagnosed [62]. Many delirium screening tools have been developed, such as the Confusion Assessment Method for the ICU (CAM-ICU) [63] and the Intensive Care Delirium Screening Checklist (ICDSC) [64]. In our hospital, we use the CAM-ICU to monitor delirium, because it can be completed in less than 1 minute and the pooled values for sensitivity and specificity are 80% and 95.9%, respectively, although the measure has several limitations [64].

Delirium and dementia frequently coexist in older patients. Dementia is a leading risk factor for delirium and delirium is an independent risk factor for subsequent development of dementia [65]. The differentiation between delirium and dementia is of crucial importance, as their assessment and clinical management are distinct. There are many signs and symptoms that can differentiate the two conditions, such as onset, duration, consciousness, speech, and causes [66].

Multicomponent non-pharmacological risk factor approaches are the most effective strategies to prevent delirium, and include discontinuation or dose reduction of anticholinergic and psychoactive drugs; family or companion involvement for reorientation and comfort; non-pharmacological approaches to sleep and relaxation; creation of a quiet, soothing, warm environment; and pain management [65, 67]. There is no convincing evidence that pharmacological prevention or treatment is effective for this condition. Drugs should be used only in severely agitated patients for whom interruption of essential medical therapies or self-harm is a risk, or in patients with extremely distressing psychotic symptoms [68].

## Enteral nutrition: It is important especially for malnourished VOPs

Malnutrition affects 12% to 45% of hospitalized older patients [69]. In critically ill patients, malnutrition and negative protein-energy balance are associated with longer ICU length of stay, mortality, rate of acquired infection, and duration of mechanical ventilation [70, 71]. Several diseases and impairments may directly influence the balance between nutritional needs and intake, including age, frailty, cognitive and physical decline, depressive symptoms, emotional variations, poor oral health, and socioeconomic changes [72, 73]. Even in cases of adequate nutrient and energy intake, the nutritional status of older adults can be impaired by compromised nutrient metabolism, drug-nutrient interactions, or altered nutrient needs [74].

Structured assessment and documentation of patient nutritional status are needed to ensure sufficient attention and intervention regarding nutrition. The Subjective Global Assessment (SGA), the Mini Nutritional Assessment (MNA), and the Geriatric Nutrition Risk Index (GNRI) are nutritional assessment tools developed specifically for elderly people; however, there is no single tool that can be considered the universal gold standard for diagnosis [69].

The feeding route can modulate splanchnic flow, as demonstrated in a study by Gatt et al. Postprandial superior mesenteric artery blood flow substantially increased with oral and enteral feeding, but decreased with parenteral feeding [75]. As oral intake is often impossible in older patients in the ICU, enteral nutrition is the recommended feeding route; however, it is often unable to fully provide nutritional needs [76, 77]. Parenteral nutrition is recommended if enteral nutrition is not feasible [70].

## Frailty: A risk factor that should not be neglected

It is very important to be able to predict which VOPs will benefit from ICU admission and have a chance of long-term survival with good quality of life. In general ICU patients, severity of illness and comorbidities at admission and treatment limitations are associated with outcome [49]; however, in VOPs, the concept of frailty seems to be more important than illness severity or traditional comorbidity measures [78]. Frailty is a clinical state of increased susceptibility owing to age-associated decline in reserve and function in a wide range of physiological systems. The concept is increasingly incorporated into prediction models to adjust for the effect of biological age as opposed to chronological age. Common signs and symptoms of frailty include fatigue, weight loss, weakness, low activity level, slow motor performance, and cognitive loss [79].

Frailty is a disorder that involves several inter-related physiological systems; the brain, endocrine system, immune system, and skeletal muscles are the organ systems that have been most studied in research on the development of frailty [80]. Fried et al found a nonlinear relation between the number of abnormal systems and frailty, independent of age and comorbidity, and the number of abnormal systems was more predictive of frailty than abnormalities in any particular systems[81].

There is currently no consensus on the best approach to screen or identify patients with prehospital frailty on admission to the ICU setting. The Clinical Frailty Scale (CFS), the Frailty Index (FI), and the Frailty Phenotype (FP) are common scales used to predict outcomes. The CFS views frailty as a multidimensional risk state that ranges from 1 (very fit) to 9 (terminally ill); scores of 5 to 8 indicate frailty. In a study of 5132 critically ill patients aged ≥80 years, frailty (CFS score >4) was present in 43.1% and was independently related to ICU admission (22.2%) and 30-day mortality (35.8%) [82]. The FP assumes that frailty is a biological syndrome resulting from cumulative decline across multiple physiological systems, and contains five criteria (shrinking, weakness, slowness, low-level physical activity, and self-reported exhaustion). An FP score ≥3 is a risk factor for ICU mortality [83]. The perfusion index, which measures health deficit accumulation, may also help to improve critical care outcome prediction in older adults [84]. A meta-analysis of 10 observational studies and 3030 ICU patients showed that frailty is associated with higher hospital mortality and long-term mortality, and that frail patients are less likely to be discharged home than fit patients [85].

## Geriatrician care: Comprehensive evaluation is helpful for the rehabilitation of VOPs

Geriatric expertise is usually not available on a regular basis in other wards, and it is not usual for a geriatrician consultation to be sought when deciding whether to discharge an elderly patient [86]. However, owing to their expertise in multimorbidity and acute stress in older patients, geriatricians can provide a more comprehensive assessment of older patients that may lead to better care and orientation decisions for those patients. Recently, geriatrician involvement has been found to provide a survival benefit in many other groups; furthermore, specialized geriatric ICU care may greatly improve survival in older adults, compared with general ICU patients [84]. One small-scale, single-center study illustrated the benefits of geriatric intervention for functional dependency in 45 older patients after ICU discharge, and showed that physical function was usually recovered rapidly [87].

Including a geriatrician in shared decision making for critically ill older patients may improve their outcomes. However, there are no large-scale studies that support this hypothesis. The effect of including a geriatrician in the assessment of octogenarians in the ICU still needs to be demonstrated using randomized trials [88].

## Homeostasis: The cornerstone of treatment for critically ill VOPs

In elderly patients, physiological changes associated with aging increase the likelihood of fluid and electrolyte disorders [89]. The most important changes are a decrease in glomerular filtration rate, decreased urinary concentrating ability, and narrowed limits for the excretion of water, sodium, potassium, and acid. Critically ill older patients may lose the ability to maintain homeostasis, making them more susceptible to hyponatremia, hypernatremia, volume depletion, volume overload, hyperkalemia, and metabolic acidosis. Therefore, it is important to be aware of potential electrolyte abnormalities in elderly patients to prevent adverse outcomes.

Hypernatremia and hyponatremia are the most common electrolyte abnormalities in elderly patients, and both are associated with high mortality rate[90]. Delirium, seizures, and coma are common symptoms of hyponatremia [91], and consciousness disorders and confusion can be associated with hypernatremia [92]. Owing to the widespread administration of fluids or diuretics in the ICU, hypernatremia is more common, especially in elderly patients [93]. Extreme care must be taken to avoid excessively rapid correction or overcorrection of hypernatremia, as it increases the risk of iatrogenic cerebral edema, with possibly serious consequences. Many medications prescribed to elderly patients can interfere with urinary excretion of potassium. It must be recognized that both hypokalemia and hyperkalemia are associated with life-threatening cardiac arrhythmia, and the risks and benefits of using large doses of diuretics or potassium-sparing agents should be weighed cautiously when making treatment decisions.

Alterations in calcium, magnesium, and phosphorus homeostasis are also very common in elderly critically ill patients. Concentrations of these electrolytes tend to be elevated owing to renal insufficiency. However, poor nutrition intake and use of some medications can lead to deficiency states. Therefore, levels of these electrolytes should be carefully monitored in elderly critically ill patients.

## Infection: Prevention and early recognition are more important

A national survey conducted in China in 2018 showed that sepsis-related mortality accounted for up to 12.6% of total mortality. In VOPs, the mortality rate is nearly 50 times that of their younger adult counterparts (3136.5 vs. 66.7 per 100,000 population, p<0.0001) [50]. Hospital-acquired infection in VOPs is life threating and preventable. Drawing on our experiences, we will summarize the predisposing factors in VOPs in terms of open access owing to intubation and immune senescence.

Many mechanisms account for immune senescence, including alterations in the mucosal lining barriers [94], loss of the proliferative capacity of immune cells [95], impaired signal transduction, and decreased antibody response [96-98]. Moreover, the risk of infection in VOPs increases sharply on hospitalization, because of the use of tubes [99, 100]. The 2017 Extended Prevalence of Infection in Intensive Care (EPIC) III study, which was conducted worldwide, showed that the most common sites of infection in ICUs are the respiratory tract, abdomen, and bloodstream [101], mainly owing to mechanical ventilation, impaired intestinal barrier, and catheterization. Of infections in VOPs, ventilation-associated pneumonia, urinary tract infections, and catheter-related bloodstream infections are preventable and controllable. In our hospital, constant evaluation of hand hygiene compliance, strict assessment of sterile operations, and airway management have reduced ventilation-associated pneumonia and catheter-related bloodstream infections infection rates by 90%. It is also important to remove all unnecessary catheters as early as possible. Another cause of iatrogenic infection is the overuse of antibiotics. *Clostridium difficile* infection is a typical example. our ward has set up an antibiotic guidance and monitoring group to improve the use of antibiotics.

Care strategies for ICU patients must take into account the unique characteristics of VOPs. First, they require early identification of infection. VOPs may not show infection-specific symptoms or signs. Low-grade temperature elevation, increased confusion, and anorexia may be the earliest signs, though they do not have a high positive predictive value [102]. For some elderly patients, exacerbation of underlying diseases (e.g., AF) may be a major sign of infection. Thus, comprehensive assessment of the underlying condition helps to identify the infection early. Second, the simple principle of “start low, go slow” applies to the administration of most drugs, but not to antibiotics. Some antibiotics with concentration-dependent activity work best when the drug concentration is well above the minimum inhibitory concentration, which is more important for successful treatment in older adults than in younger adults [103, 104]. For VOPs with severe infections, it is important to use the highest dose within a safe range when administering antibiotics for the first time.

From the end of 2019, a pandemic caused by severe acute respiratory syndrome coronavirus 2 (SARS-CoV-2), termed COVID-19, has spread globally. Older adults are more likely to have severe disease. A study based on data from mainland China found that the hospitalization rate for COVID-19 increased with age, from 1% for individuals aged 20-29 years to 18% for those aged >80 years [105]. Mortality is also associated with older age. A report from the Chinese Center for Disease Control and Prevention showed that case fatality rates are 8% for individuals aged 70-79 years and 15% for those aged ≥80 years [106]. In the United States, 80% of deaths occur in individuals aged ≥65 years [107]. This may be related to the poor immune function of older adults, which weakens their response to pathogens [108]. Age, Sequential Organ Failure Assessment (SOFA) score, Acute Physiology and Chronic Health Evaluation (APACHE) score, platelet count <125 × 10^9^/L, d-dimer, creatinine >133 μmol/L, interleukin-6, and lung consolidation on admission are independent risk factors for disease severity among older patients with COVID-19 [109]. Older age is a relative contraindication for the use of some advanced life support equipment, such as extracorporeal membrane oxygenation. Therefore, the key treatment strategy principle for these patients after COVID-19 diagnosis is to provide aggressive support therapy (e.g., respiratory support and nutrition support) to prevent deterioration, rather than treat the condition when it becomes severe. Furthermore, strengthening protection and control strategies for older adults is a priority.

This paper has focused on systemic therapy for VOPs. However, whether VOPs can benefit from ICU care remains controversial [110], and there is no consensus among clinicians on the triage process [111]. This is the main therapeutic difference between VOPs and younger adult patients. During the COVID-19 pandemic, doctors have often taken old age into consideration when deciding on the provision of life-sustaining measures, and conservative treatment plans are generally adopted for elderly patients. In most countries, decisions about whether to administer risky or traumatic procedures such as tracheal intubation mainly depend on the patient’s living will and family choices [112]. A systematic review concluded that age and illness severity were most strongly associated with ICU admission refusal, especially during periods of reduced bed availability [113]. Advanced age is an independent risk factor for do-not-resuscitate orders. Whether this is ethical needs to be explored in depth.

Age alone does not predict mortality, even in the most vulnerable VOPs. The most important factor in determining if ICU admission is appropriate for an elderly patient is to consider whether or not intensive care is individualized and comprehensive. The ABCCDEFGHI bundles summarize our thoughts and suggestions about intensive care for critically ill VOPs and may provide new ideas for the diagnosis and comprehensive management of VOPs during clinical practice.
